# Supplementary material for: The Relationship Between Task‐Related Aperiodic EEG Activity, Neural Inefficiency and Verbal Working Memory in Younger and Older Adults
Source: Psychophysiology. 2026 Jan 30;63(2):e70255. doi: 10.1111/psyp.70255 (PMC12856785; doi:10.1111/psyp.70255)
Supplement: Supplementary file 1 — Figure S1: Median aperiodic exponent during task segments (F—fixation, R—retention) for younger (orange) and older (navy) adults across ROIs. Exponent is the average of all WM loads (load‐1, load‐3 and load‐5). Error bars represent 95% credible intervals based on the highest density interval (HDI). Figure S2: The distribution of individual participant slopes used to calculate the neural inefficiency scores reported in Section 3.2. These distributions provide information about the underlying linear relationships between P3b amplitude, WM performance, and WM load that form the basis of the neural inefficiency measure. Figure S2: Individual participant slopes for (A) P3b amplitude during encoding, (B) P3b amplitude during probe presentation, (C) sensitivity (dL), and (D) response time (RT) as a function of WM load. Slopes were calculated by regressing each measure against WM load (1, 3, 5) for each participant separately. Histograms show the distribution of raw slopes for younger (blue) and older (orange) adults. Dashed vertical lines indicate zero slope (no change with load). Figure S3: Relationship between pre‐stimulus aperiodic exponents and neural inefficiency across age groups and ROIs. Neural inefficiency scores calculated during encoding (A–C) and probe phases (D–F) are plotted against aperiodic exponents measured during fixation (top row) and the final retention period R4 (bottom row), respectively. Each column represents the following ROIs: frontal (A, D), central (B, E), and parieto‐occipital regions (C, F). Orange dots represent younger adults, and blue dots represent older adults. Gray lines show the estimated linear fit across all participants, and shaded gray represents the 95% HDI for the fit. [file PSYP-63-e70255-s001.docx]

**Supplementary Results**

*S1 Regional Differences*

The model with ROI added as a fixed effect demonstrated differences in aperiodic exponents across regions, with frontal regions showing lower exponents compared to central (median difference = -0.16 [-0.27, -0.06], *pd* = 99.75%, 0% in ROPE), but not parieto-occipital regions (median difference = 0.01 [-0.10, 0.11], pd = 60.85%, 51.89% in ROPE). There was evidence for an interaction between age group and parieto-occipital ROI (median difference = 0.05 [0.01, 0.08], *pd* = 99.62%, 26.47% in ROPE), and for younger adults having distinct changes in frontal regions during later retention periods compared to older adults (R3: *pd* = 98.85%, 23.92% in ROPE; R4: *pd* = 98.22%, 18.55% in ROPE). Similar effects were observed in parieto-occipital regions during R3 (*pd* = 98.75%, 23% in ROPE), however, practical relevance was inconclusive in all interactions. No effects involving WM load reached the threshold for probable directional evidence or practical relevance (all *pd* < 94.70%, > 26.47% in ROPE).

Post-hoc comparisons examining age differences across segments and ROIs revealed that while young adults consistently showed larger exponents than older adults, the magnitude of this difference varied by ROI and task stage. Central and parieto-occipital regions showed practically relevant age differences across all segments (all *pd* > 99.92, 0% in ROPE) (Figure 2B-C). In contrast, frontal regions showed strong age differences at fixation (*pd* = 99.92%, 0% in ROPE) that diminished during late retention (R3/R4: *pd* > 97.23%, > 4.89% in ROPE; Figure 2A).

**

Figure S1. Median aperiodic exponent during task segments (F – fixation, R – retention) for younger (orange) and older (navy) adults across ROIs. Exponent is the average of all WM loads (load-1, load-3 and load-5). Error bars represent 95% credible intervals based on the highest density interval (HDI).

*S2 Neural Inefficiency*

*S2.1 Neural Inefficiency Slope Distributions*

Figure S2 shows the distribution of individual participant slopes used to calculate the neural inefficiency scores reported in Section 3.2. These distributions provide information about the underlying linear relationships between P3b amplitude, WM performance, and WM load that form the basis of the neural inefficiency measure.

Most participants showed positive P3b slopes at encoding (younger: M = 0.07, SD = 1.47; older: M = 0.36, SD = 1.29), indicating P3b amplitude increased with WM load. At the probe, P3b slopes were predominantly negative (younger: M = -0.32, SD = 2.82; older: M = -0.68, SD = 0.94), reflecting the typical pattern where P3b amplitude decreases as task difficulty increases during the response phase. dL slopes were near zero for both groups (younger: M = -0.18, SD = 1.35; older: M = -0.02, SD = 1.84), reflecting the ceiling effects in behavioural performance on average, but with some individual heterogeneity. RT slopes were positive for both groups (younger: M = 0.10, SD = 0.09; older: M = 0.14, SD = 0.16), confirming that response times increased with WM load as expected.

**

Figure S2. Individual participant slopes for (A) P3b amplitude during encoding, (B) P3b amplitude during probe presentation, (C) sensitivity (dL), and (D) response time (RT) as a function of WM load. Slopes were calculated by regressing each measure against WM load (1, 3, 5) for each participant separately. Histograms show the distribution of raw slopes for younger (blue) and older (orange) adults. Dashed vertical lines indicate zero slope (no change with load).

*S2.2 Regional Differences in Neural Inefficiency Relationships*

*S2.2.1 Encoding*

To understand regional effects, separate models were run using the fixation exponent derived from each ROI. The models revealed robust evidence for a relationship between greater neural inefficiency with smaller (i.e. flatter) fixation exponents in both frontal (median = -2.59 [-4.80, -0.29], *pd* = 98.78%, 0% in ROPE) and central regions (median = -3.23 [-5.96, -0.73], *pd* = 99.20%, 0% in ROPE), but not in the parieto-occipital region (median = -1.10 [-3.57, 1.40], *pd* = 81.15%, 5.87% in ROPE) (Figure S3A-C). In all models, there was little evidence for an interaction between age group and the fixation exponent on neural inefficiency (all *pd* < 85.52, all > 4.50% in ROPE).

However, ROI-specific models revealed no evidence for relationships between the fixation exponent and RT neural inefficiency (all *pd* < 92.55%, > 2.00% in ROPE).

*S2.2.2 Probe*

ROI based models demonstrated evidence for a relationship between R4 exponent in the central region and neural inefficiency to the probe, with smaller exponents associated with greater neural inefficiency (median = -2.69 [-4.71, -0.59], *pd* = 99.35%, 0% in ROPE) (Figure 5G). For the frontal region, there was a trend towards a relationship between the R4 exponent and neural inefficiency (median = -1.61 [-3.59, 0.35], *pd* = 94.47%, 3.05% in ROPE), but this did not meet the threshold for a probable effect (Figure 5F), while no consistent effect was seen for the parieto-occipital region (median = -0.64 [-2.66, 1.17], *pd* = 75.12%, 9.21% in ROPE) (Figure 5H). In all models, there was little evidence for an interaction between age group and R4 exponent on probe neural inefficiency (all *pd* < 83.17%, all > 4.68% in ROPE).

For RT neural inefficiency, directional evidence trended towards a relationship between the R4 exponent and neural inefficiency in all ROIs, these did not meet the threshold for a probable effect (all *pd* < 86.20%, > 3.39% in ROPE).

Figure S3. Relationship between pre-stimulus aperiodic exponents and neural inefficiency across age groups and ROIs. Neural inefficiency scores calculated during encoding (A-C) and probe phases (D-F) are plotted against aperiodic exponents measured during fixation (top row) and the final retention period R4 (bottom row), respectively. Each column represents the following ROIs: frontal (A, D), central (B, E), and parieto-occipital regions (C, F). Orange dots represent younger adults, and blue dots represent older adults. Grey lines show the estimated linear fit across all participants, and shaded grey represents the 95% HDI for the fit.
